# Stillbirth surveillance and review in rural districts in Bangladesh

**DOI:** 10.1186/s12884-018-1866-2

**Published:** 2018-06-13

**Authors:** Abdul Halim, Mamuda Aminu, Juan Emmanuel Dewez, Animesh Biswas, A. K. M. Fazlur Rahman, Nynke van den Broek

**Affiliations:** 1 Centre for Injury Prevention and Research Bangladesh (CIPRB), Dhaka, Bangladesh; 2 Centre for Maternal and Newborn Health, Liverpool School for Tropical Medicine, Pembroke Place, Liverpool, L3 5QA UK; 3 Örebro University, Örebro, Sweden

**Keywords:** Stillbirth, Surveillance, Cause of death, Care seeking, Verbal autopsy, Bangladesh

## Abstract

**Background:**

An estimated 2.6 million stillbirths occur every year, with the majority occurring in low- and middle-income countries. Understanding the cause of and factors associated with stillbirth is important to help inform the design and implementation of interventions aimed at reducing preventable stillbirths.

**Methods:**

Population-based surveillance with identification of all stillbirths that occurred either at home or in a health facility was introduced in four districts in Bangladesh. Verbal autopsy was conducted for every fifth stillbirth using a structured questionnaire. A hierarchical model was used to assign likely cause of stillbirth.

**Results:**

Six thousand three hundred thirty-three stillbirths were identified for which 1327 verbal autopsies were conducted. 63.9% were intrapartum stillbirths. The population-based stillbirth rate obtained was 20.4 per 1000 births; 53.9% of all stillbirths occurred at home. 69.6% of mothers had accessed health care in the period leading up to the stillbirth. 48.1% had received care from a highly trained healthcare provider. The three most frequent causes of stillbirth were maternal hypertension or eclampsia (15.2%), antepartum haemorrhage (13.7%) and maternal infections (8.9%). Up to 11.3% of intrapartum stillbirths were caused by hypoxia. However, it was not possible to identify a cause of death with reasonable certainty using information obtained via verbal autopsy in 51.9% of stillbirths.

**Conclusions:**

Introducing surveillance for stillbirths at community level is possible. However, verbal autopsy yields limited data, and the questionnaire used for this needs to be revised and/or combined with information obtained through case note review.

Most women accessed and received care from a qualified healthcare provider. To reduce the number of preventable stillbirths, the quality of antenatal and intrapartum care needs to be improved.

**Electronic supplementary material:**

The online version of this article (10.1186/s12884-018-1866-2) contains supplementary material, which is available to authorized users.

## Background

Of the estimated 2.6 million stillbirths that occur each year, the majority occur in low- and middle-income countries and at least half of all stillbirths are preventable [1]. However, in most low- and middle-income countries (LMIC), vital registration systems are not in place and the true number of stillbirths that occur is not known. In 2014, the World Health Organization (WHO) and the United Nations Children’s Fund (UNICEF) launched the Every Newborn Action Plan (ENAP), a road map to reduce preventable neonatal deaths and stillbirths [2]. One of the recommendations of the ENAP is to improve the recording of every birth, neonatal death, and stillbirth. Improved surveillance should provide contemporaneous data, help to continue to raise awareness and should lead to renewed action and implementation of evidence-based interventions to prevent these deaths wherever possible. ENAP stipulates a target of less than 10 stillbirths per 1000 births by 2035 [2].

Bangladesh is a unique example in terms of health gains despite presenting poorer development indicators than other South Asian countries [3]. Bangladesh showed the fastest annual reduction in stillbirth rates among all countries in South Asia over the 2000–2015 period [1]. Notwithstanding this important reduction, Bangladesh is still ranked seventh globally in terms of absolute number of stillbirths, with an estimated 83,000 stillbirths per annum and a stillbirth rate of 25.4 per 1000 births [4].

Perinatal death surveillance and review is an effective strategy for obtaining data that can be used to improve the quality of care delivered to women and their babies [5, 6]. Perinatal death reviews help to reduce perinatal morbidity and mortality by providing information on the major causes of, and factors contributing to, stillbirths and neonatal deaths for more targeted and effective action. The cause of most stillbirths in LMIC is never established [7]. A systematic review of the literature highlights the need for more and more accurate data on cause of and factors contributing to stillbirth in LMICs. Most studies are low-quality, hospital-based studies, most of which focused on causes related to maternal diseases, such as hypertensive disorders and infections, and fetal congenital anomalies and do not reflect cause of stillbirth in the general population [8].

The Government of Bangladesh implemented a population-based Maternal and Perinatal Death Review (MPDR) system in four rural districts of the country in 2011. This study was conducted to assess the feasibility of implementing such a population-based surveillance system for stillbirths and to use verbal autopsy to document the cause of and factors contributing to stillbirths occurring either at facility level or in the community.

## Methods

### Study area

The four target districts, Jamalpur, Moulvibazar, Narail and Thakurgaon, comprising a total population of 6.7 million, were targeted based on their relatively poor maternal and neonatal health indicators: uptake of antenatal care (ANC) (63.5% versus 67.7% nationally) and percentage of deliveries attended by a skilled provider (19.9% versus 31.4% nationally) [9]. In addition, these districts were the target districts for the joint Government-UN Maternal Newborn Health Initiative which focused on saving maternal and neonatal lives through improved district level planning, investments in infrastructure and supplies and also strengthening of human resources.

### Identification of stillbirths

All grassroot level health and family planning workers, Health Assistants (HA) and Family Welfare Assistants (FWA) (each responsible for a population of 5000–6000) were trained to identify all stillbirths at household level in their area using the following definition of stillbirth: “Birth of a baby after 28 weeks of gestation and who showed no evidence of life, such as beating of the heart, pulsation of the umbilical cord, or definite movement of voluntary muscles, whether or not the umbilical cord has been cut or the placenta is attached, immediately after birth” [10]. This definition also conforms with the WHO definition of stillbirth for international comparison [11].

Trained HAs or FWAs used a network of local community members (teachers, social workers, elected community women members, community health workers, and traditional birth attendants) to obtain information on any stillbirth which occurred in their assigned area. After receiving notification about a stillbirth, the HA/FWA made a household visit to confirm that the death met the definition criteria. If the definition criteria were fulfilled, the HA/FWA completed a stillbirth notification slip. The slips were sent to an assigned focal person at each *upazila* (sub-district administrative centre) within 7 to 15 days after the death.

### Verbal autopsy

An expert, multi-disciplinary team under the guidance of the Directorate General of Health Services and the Directorate General of Family Planning (DGFP) developed a verbal autopsy (VA) questionnaire based on recommended WHO VA tools and the existing neonatal death audit forms already available in Bangladesh [12, 13]. The questionnaire was adapted for use by district healthcare workers and family planning workers and translated into Bangla (Additional file 1). The VA questionnaire was then field tested in one district before use in all four study districts. The VA questionnaire included 29 close-ended questions with different response categories including questions on sociodemographic characteristics of the mother, complications during pregnancy and childbirth, access to antenatal care, mode of delivery, healthcare seeking behaviour at the time of antepartum or intrapartum complications, and characteristics of the stillbirth.

To assess if a stillbirth was fresh (likely intrapartum death) or macerated (likely antepartum death), questions around the appearance at birth and whether or not mothers had felt the baby move were included in the VA questionnaire.

Field level supervisors of HAs and FWAs (i.e. Health Inspectors (HI), Assistant Health Inspectors (AHI), and Family Planning Inspectors (FPI)) who oversee a population of 25,000–30,000, received training on the use of the VA questionnaire, facilitated by a team of trained social scientists and medical doctors. Each supervisor conducted at least five VA under supervision to ensure competency in using the tool.

**Fig. 1:**
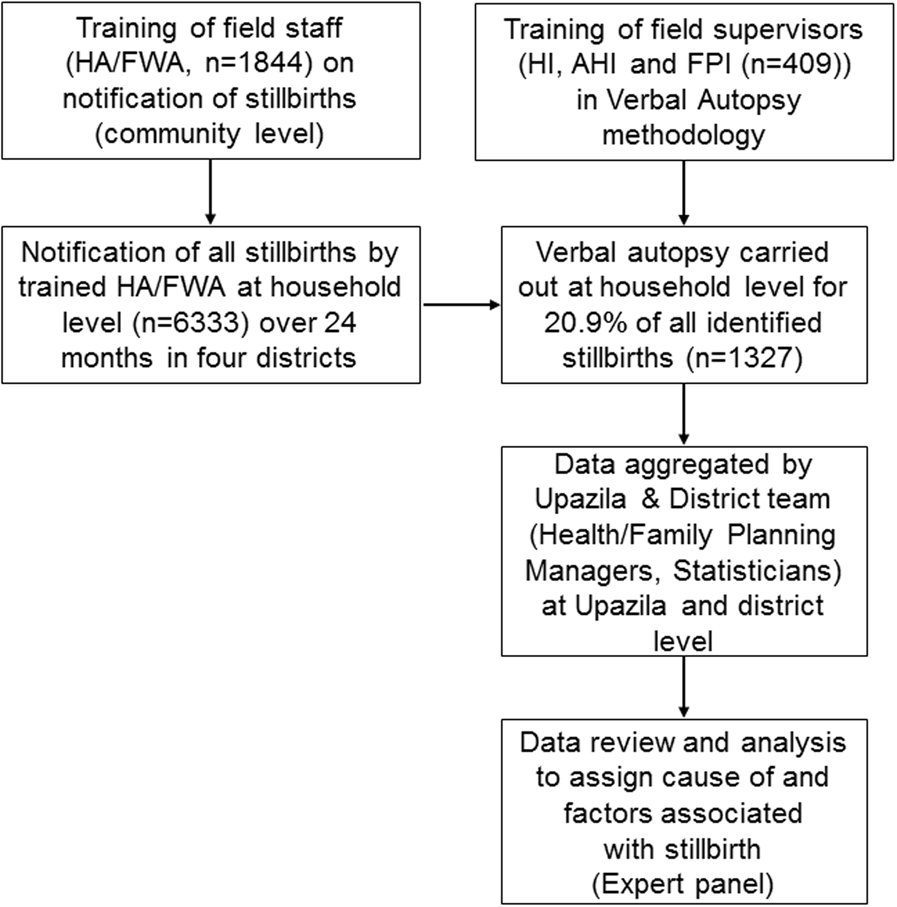
Process of stillbirth surveillance (notification) and review (using verbal autopsy) in four districts in Bangladesh. AHI = Assistant Health Inspectors, FPI = Family Planning Inspectors, FWA = Family Welfare Assistant, HA = Health Assistant, HI = Health Inspector

Upon receipt of a death notification, a trained field level supervisor was assigned to conduct a VA at household level of every fifth stillbirth (identified sequentially) which occurred in his/her assigned area. Each VA interview had to include three respondents including the mother, father or relatives who were present at the time of the stillbirth (Fig. 1).

### Data analysis

Sociodemographic characteristics of the stillbirths and mothers as well as the circumstances around the death were described using frequencies and proportions.

### Assigning cause of death

An expert panel was created, comprising of an obstetrician, a physician experienced in stillbirth classifications and a paediatrician, to develop a hierarchical model to assign cause of stillbirth. The model was based on information obtained from a systematic literature review to determine cause of and factors associated with stillbirth in LMIC [8]. We considered all causes with characteristics which could potentially be identified using verbal autopsy (VA). Causes with the strongest available evidence to support the diagnosis were ranked first. Eight causes of stillbirth were identified: eclampsia, hypertension, antepartum haemorrhage, diabetes, infection, intrapartum-related hypoxia, external trauma, and ruptured uterus. A category “unknown” was used for any case where there was insufficient or no information to be able to assign a cause of death.

We developed computer-based algorithms containing the specified characteristics of each condition to assign cause of stillbirth for each identified case of stillbirth for which verbal autopsy was conducted. Criteria for diagnosis were developed and agreed with input from all researchers, following a hierarchical model (Additional file 2). For each diagnosis, we considered how likely it is for the diagnosis to be accurate using data obtained via verbal autopsy only. For example, a history of hypertension with convulsion is more likely to be an accurate indication of eclampsia than a history of fever and jaundice for the diagnosis of infection. The algorithm was applied independently by two researchers to all cases of stillbirth initially, and then to fresh and macerated stillbirths separately. There were no discrepancies in results obtained between the two researchers.

### Ethical approval

Ethical clearance was obtained from the Ethical Review Committee (ERC/CIPRB/2010/01) of the Centre for Injury Prevention and Research Bangladesh. The protocol and tools were reviewed and approved for implementation by the Directorate General of Health Services of Bangladesh. Informed, written consent was requested and obtained from each respondent before each verbal autopsy interview. Anonymity and confidentiality was maintained throughout the process. Participation was voluntary.

## Results

### Stillbirth rate

A total of 6333 stillbirths were identified between January 2011 and December 2012 in the study area, 3025 in year 1 and 3308 in year 2. A total of 1327 VA (20.9% of all stillbirths reported) were performed.

Using the 2011 crude birth rate of Bangladesh of 22.6 livebirths per 1000 population [13], we estimated a total of 151,420 live births in the study area per year giving an estimated stillbirth rate of 19.6 and 21.4 per 1000 births in 2011 and 2012, respectively.

### **Maternal characteristics** (Table 1)

The majority of the VA conducted (65%) included the mothers of the babies who had been stillborn. The mean (SD) age of the mothers was 23.7 (±5.7) years. Most of the mothers (1032, 77.7%) had at least completed primary education and 283 (21.3%) had completed secondary education or above.

**Table 1:** Characteristics and sociodemographic information of women who experienced a stillbirth (*n* = 1327)

Characteristics		
Age categories (years)	n	%
15–19^a^	310	23.3
20–24	491	37.0
25–29	284	21.4
30–34	147	11.1
35 and above	95	7.2
Education level		
No schooling received	26	2.0
Primary incomplete	269	20.3
Primary complete	228	17.2
Secondary incomplete	478	36.0
Secondary or higher	283	21.3
Do not know	43	3.2
Parity		
1	568	42.8
2 to 4	648	48.8
5 or more	111	8.4
Reported pregnancy duration (months)		
7–8	421	31.7
9	802	60.4
> 9	104	7.8
Antenatal care during pregnancy		
Any ANC	1059	79.8
Four or more ANC visits	449	33.8
ANC provided by skilled provider (Doctor, Medical Assistant, Nurse-midwife)	761	57.3
Reported complications during pregnancy or delivery		
Complication during pregnancy	1041	78.4
Complication during delivery	865	65.2
Mode of delivery		
Normal Vaginal delivery	1147	86.4
Assisted vaginal delivery (ventouse or foreps)	33	2.5
Caesarean section	142	10.7
Place of delivery		
At home	715	53.9
En route to a health facility	33	2.5
At a health facility	579	43.6
Birth attendant at delivery		
Skilled birth attendant (Doctor, Medical Assistant, Nurse-midwife)	614	46.3
Unskilled attendant/family member/untrained TBA)	713	53.7

^a^ No women were less than 15 years of age

Five hundred sixty-eight (42.8%) of mothers were primiparous. The majority (802, 60.4%) reported delivery of a term stillbirth, while 421 (31.7%) reported a preterm delivery and 104 (7.8%) a post-term.

### Pregnancy and delivery characteristics

Most mothers (1059, 79.8%) attended ANC at least once during the index pregnancy and 449 (33.8%) attended four or more times (Table 1). Almost 6 in 10 (57.3%) had received care from a skilled healthcare provider (a doctor, a nurse / midwife or a medical assistant).

More than half of all stillbirths (715 (53.9%)) were born at home or at the house of a traditional birth attendant. Most of the mothers had a normal vaginal delivery (1147, 86.4%). Overall, 10.7% of stillbirths were delivered by Caesarean section and 2.5% by assisted vaginal delivery (vacuum or forceps) or were a breech delivery. 614 (46.3%) of the deliveries were assisted by skilled healthcare providers.

### Complications during pregnancy and delivery

During VA, 1041 (78.4%) of the mothers reported at least one type of complication during pregnancy (antepartum) and 865 (65.2%) reported at least one type of complication at time of birth (Table 2).

**Table 2:** Complications during pregnancy and childbirth as reported by mothers who had experienced a stillbirth (*n* = 1041)

Antepartum Complications	Frequency (*n* = 865)	%
Hypertension	202	19.4
Facial or limb oedema	384	36.9
Blurred vision	435	41.8
Convulsions (eclampsia or epilepsy)	137	13.2
Unconsciousness	148	14.2
Antepartum Haemorrhage	223	21.4
High fever	470	45.1
Diabetes	101	9.7
Anaemia	414	39.8
Jaundice	99	9.5
Other complication (not specified)	294	28.2
Intrapartum Complications	Frequency (n = 865)	%
PROM – Premature rupture of membranes	454	52.4
Long labour (> 12 h)	477	55.1
Obstructed labour	315	36.4
Mal-presentation	166	19.2
Convulsions (eclampsia or epilepsy)	100	11.6
Haemorrhage	262	30.3
Retained placenta	100	11.6
Others (unspecified)	136	15.7

Only 11% of women had not recognised nor reported a complication during pregnancy or at the time of birth. This was similar for both antepartum and intrapartum.

### Care seeking for antepartum complications

**Fig. 2:**
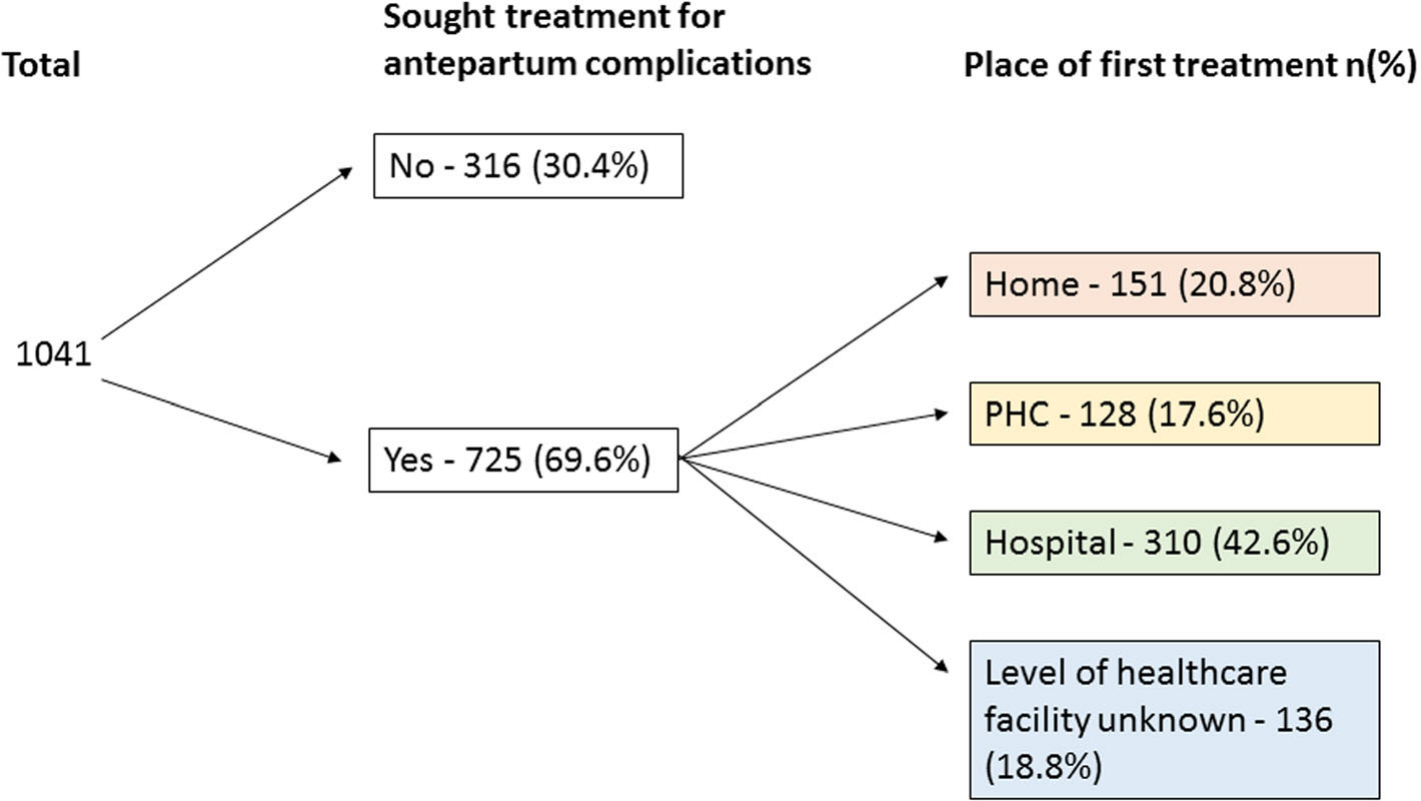
Care seeking pattern for women who had a recognised complication during pregnancy (*n* = 1041). PHC = Primary Healthcare Centre

Among the 1041 mothers who experienced an antepartum complication, 725 (69.6%) had sought care. Among them, 438 (60.4%) accessed care at a health facility. The healthcare facility was a secondary or tertiary level of care in 310 (42.6%) of the cases (Fig. 2).

Among the 725 women who sought care for antepartum complications, 349 (48.1%) received care from a medical doctor trained in identifying and treating most antenatal complications.

### Characteristics of stillbirths

The sex of the stillborn babies was known in 928 (69.9%) of the cases. Of these, 534 (57.5%) were males. A total of 272 (20.5%) were described by mothers as “smaller than normal at birth”.

At the time of verbal autopsy, 848 (63.9%) of the stillbirths were reported to be fresh stillbirths and 464 (34.9%) macerated at time of birth. However, when considering stricter criteria (including only those who had reported fetal movement at onset of labour and were considered to look like fresh stillbirths), only 491 cases (58.3%) could be reported as intrapartum stillbirths and 351 (41.7%) as antepartum stillbirths.

### Likely cause of stillbirth

Of the 1327 cases of stillbirth, 66 (5%) were due to eclampsia, 136 (10.2%) to hypertension, 182 (13.7%) to antepartum haemorrhage, 41 (3.1%) to diabetes, 115 (8.7%) to infections, 96 (7.2%) to intrapartum-related hypoxia (only those without antepartum haemorrhage, hypertension or eclampsia identified – the hierarchical model), and 3 (0.2%) due to external trauma. No cases of ruptured uterus were reported. No cause could be assigned in 688 (51.9%) of the cases (Table 3; Additional file 3). There was no significant difference between the distribution of likely cause of death for fresh compared to macerated stillbirths (*p* = 0.444), after accounting for intrapartum-related hypoxia, which may not be a cause of death in macerated stillbirths.

**Table 3:** Likely cause of stillbirth as assigned by expert panel using hierarchical model

Likely Cause of Stillbirth	Type of Stillbirth		All Stillbirths Combined n (%)
	Fresh Stillbirth n (%)	Macerated Stillbirth n (%)	
Eclampsia	39 (4.6)	27 (5.8)	66 (5.0)
Hypertension	87 (10.3)	44 (9.5)	136 (10.2)
Antepartum Haemorrhage	113 (13.3)	69 (14.9)	182 (13.7)
Diabetes	20 (2.4)	21 (4.5)	41 (3.1)
Maternal Infection	77 (9.1)	38 (8.2)	115 (8.7)
Intrapartum-related hypoxia	96 (11.3)	0 (0.0)	96 (7.2)
Trauma	2 (0.2)	1 (0.2)	3 (0.2)
Unknown	414 (48.8)	264 (56.9)	688 (51.9)
Total	848 (100.0)	464 (100.0)	1327^a^ (100.0)

^a^Total includes 15 stillbirths for which it was not possible to assess if it was a fresh or macerated stillbirth

## Discussion

### Main findings

To the best of our knowledge, this is one of the largest studies to implement a stillbirth surveillance and review programme at population level in a low- or middle-income country. This study shows that district level surveillance of all stillbirths can be introduced successfully and provide a population-based, contemporaneous stillbirth rate using verbal autopsy. This can provide information on type of stillbirth (ante or intrapartum) and on likely cause of, and factors contributing to, stillbirth.

Community-based surveillance was implemented successfully in four districts in Bangladesh where 53.9% of identified stillbirths occurred at home. Two thirds of all mothers had accessed care during pregnancy for complications recognised by them (or their family) to require health care. Two thirds of these attended a healthcare facility and half of the mothers reported that they had received care from a highly trained healthcare provider.

Antepartum haemorrhage and hypertension or eclampsia were identified as the commonest causes of stillbirth accounting for almost 30% of stillbirths. Maternal infections were the third most common cause of stillbirth and identified in almost 10% of cases. However, using information obtained via verbal autopsy, did not allow the identification of a clear cause of death in half of all stillbirths. About two thirds of the cases (63.9%) were intrapartum stillbirths. There were no substantial differences in cause of stillbirth between fresh (intrapartum) and macerated stillbirths (antepartum death) except for intrapartum-related hypoxia which was present in at least 11.3% of stillbirths.

### Stillbirth rate

The stillbirth rate (SBR) obtained was 20.4 per 1000 births. The neonatal mortality rate (NMR) in the study area during the same period was 24.4 per 1000 live births [14]. Bangladesh does not have a system for vital registration data, but in this study, specific effort was made to identify stillbirths. As a proxy, we estimated the SBR/NMR ratio, which was 0.84. This is in line with high-resource settings with better civil registration systems and vital statistics. The median ratio of SBR to NMR in high income countries is 0.9 (IQR: 0.65–1.15) [4]. This may indicate that the majority of stillbirths in this study population were identified with the introduction of the population-based stillbirth surveillance.

### Cause of death

Findings should be interpreted in light of some limitations. Firstly, as cause of death was assigned hierarchically, the proportion for each cause of death could potentially change as the hierarchy changes. Secondly, verbal autopsy was used to develop a ‘clinical history’ for each case. A limitation of this is that additional clinical information that could have aided diagnosis of cause of death, including results of laboratory tests, was not available. Thus, this study allowed for the estimation of likely cause of stillbirths for those who died either at facility level or in the community in only about half of the cases (51.9%). Assigning cause of death from information obtained via verbal autopsy is known to be difficult. Studies from Bangladesh, Pakistan, Tanzania, and Ghana have reported an undetermined cause of stillbirth in 18 to 58% of cases [7, 15–19].

Furthermore, the maternal infection category does not provide information on type of infection and whether potentially preventable (such as HIV, malaria, syphilis or tuberculosis). The questionnaire used during verbal autopsy in Bangladesh also did not include questions to ascertain whether stillborn babies were identified to have had a congenital anomaly. This is because most congenital anomalies causing death are cardiovascular and chromosomal anomalies and most often cannot be detected by parents in the community [20]. In an earlier systematic review identifying causes of and contributing factors to stillbirth in low- and middle-income countries [8], the main causes of stillbirth (ordered by frequency of reporting) were maternal factors, congenital anomalies, placental causes, asphyxia, umbilical problems, and uterine factors. However, most of the studies included in the review were hospital-based, making the comparison with the results of this study difficult.

Nevertheless, in a similar study using verbal autopsy in India, Aggarwal et al. reported causes of stillbirth to include hypertension (30%), antepartum haemorrhage (16%), underlying maternal illness (12%), congenital malformations (12%) and obstetric complications (unspecified) (10%) [21]. However, in their study, stillbirths were defined as death from 24 weeks gestation and this could have explained some of the differences in cause of death observed, particularly with regard to congenital anomalies.

Baqui et al. and Nahar et al. in Bangladesh, as well as Jehan et al. in Pakistan, similarly found that maternal haemorrhage was one of the main causes of stillbirth [7, 15, 16]. Hypertensive disorders as a major cause of stillbirth was reported by Edmond et al. in Ghana [18, 19]. In Tanzania, Hinderaker et al. found that around 42% of mothers had an infection, which is four times higher than our results [17]. However, they targeted only rural communities and had a relatively small sample of 60 stillbirths compared to the sample size in this study.

To increase the proportion of cases for which a cause of death can be determined, and with a greater level of certainty, analysis of hospital records, diagnoses and management pathways would be needed. However, this would only be feasible in cases where women have been admitted to a healthcare facility and/or for whom good patient-records are available for review.

### Hierarchical model for cause of death

The variability in causes of stillbirth among studies is probably due to the use of different hierarchical models in studies [7, 18]. The proportion of any cause is dependent on the proportion of other causes and if the hierarchy of causes changes, the relative importance of any cause varies in relation to the others. In earlier studies, two different hierarchical models were applied to antepartum and intrapartum stillbirths, whereas we applied a unique model to all stillbirths. In addition, different definitions of each potential cause of stillbirth were used. For example, Hinderaker et al. defined maternal infection as “all kinds of infections” [17]**;** whereas, the definition used in this study was “the presence of fever and jaundice or fever and premature rupture of membranes”. In addition, the prevalence of malaria, the main cause of infection in the study conducted by Hinderaker et al., was higher than in our setting.

### Time of death

In terms of the proportion of ante- and intrapartum stillbirths, our finding, that the proportion of intrapartum stillbirths is higher, is in line with studies conducted in Bangladesh and Pakistan [15, 16, 22, 23]. However, Baqui et al. as well as Edmond et al. found that antepartum stillbirths represent two thirds of all stillbirths [7, 18, 19]. Intrapartum-related hypoxia was estimated to be the cause of stillbirth in 23 to 25% of cases [15, 16], which is substantially higher than the 11% we found. Another study [7], which had a lower rate of intrapartum stillbirths (37.9%), assigned intrapartum-related hypoxia as the cause of stillbirth in 20.5% of stillbirth cases.

However, half of global stillbirths occur at intrapartum period [1]. The differences observed between studies may be because, in LMIC, differentiation of antepartum and intrapartum stillbirths relies mainly on the physical appearance (fresh/macerated classification) of the stillborn, which is often not a reliable way of determining time of death. Besides it has been previously noted that healthcare providers’ assessment of physical appearance at time of stillbirth may, in fact, be an unreliable method for assessing time of death [24].

### Care-seeking behaviour

Regarding care seeking for antepartum complications, we found similar results to the study conducted by Skider et al. in a rural district of Bangladesh [25]. In both studies, more than two thirds of women had accessed and received care from a trained healthcare provider. However, the proportion of women receiving care by a trained healthcare provider was lower than in our study (30% versus 48%).

## Conclusion

Surveillance of stillbirths at community level is possible. However, using verbal autopsy to explore cause of and factors associated with stillbirth provides limited information on cause of death. It is important that if verbal autopsy is used, it should be complemented by healthcare record and case note review to establish cause of death more accurately, identify substandard care and formulate recommendations to improve quality of care and reduce preventable stillbirths.

For cases where a clear cause of death was identified, most of the causes of stillbirth were preventable. Moreover, most women had sought and received care from a qualified healthcare provider. This suggests that health care during pregnancy and around the time of birth was not optimal in most of these cases. In order to reduce the number of preventable stillbirths, more effort is needed to improve the quality of antenatal and intrapartum care.

## Additional files

Additional file 1: Questionnaire and Consent Form. (PDF 625 kb)

Additional file 2: Hierarchical model and criteria used to assign likely cause of stillbirth using information obtained via verbal autopsy. PROM = Premature rupture of membranes. (TIF 76 kb)

Additional file 3: Frequency of ante and intrapartum complications among mothers who experienced a stillbirth for all stillbirths combined, fresh stillbirths and macerated stillbirths (*n* = 1041). (TIF 95 kb)

## Abbreviations

AHI: Assistant Health Inspectors
ANC: Antenatal Care
DGFP: Directorate General of Family Planning
ENAP: Every Newborn Action Plan
FPI: Family Planning Inspectors
FWA: Family Welfare Assistants
HA: Health Assistants
HI: Health Inspectors
LMIC: Low- and middle-income countries
MPDR: Maternal and Perinatal Death Review
SBR: Stillbirth Rate
UNICEF: United Nations Children’s Fund
VA: Verbal Autopsy
WHO: World Health Organization
