# Supplementary material for: Stillbirth surveillance and review in rural districts in Bangladesh
Source: BMC Pregnancy Childbirth. 2018 Jun 13;18:224. doi: 10.1186/s12884-018-1866-2 (PMC6004696; doi:10.1186/s12884-018-1866-2)
Supplement: Supplementary file 1 — Questionnaire and Consent Form. (PDF 625 kb) [file 12884_2018_1866_MOESM1_ESM.pdf]

Government of the People's Republic of Bangladesh
Ministry of Health and Family Planning

Maternal, Still Birth and Neonatal Death Review (MPDR)

(GoB-MNH initiatives)

Still Birth review Form

(Applicable at the community)

Implementation

Directorate General of Health Services (DGHS) and
Directorate General of Family Planning (DGFP)

Technical Support

Centre for Injury Prevention and Research, Bangladesh (CIPRB)

CIPRB

UKaid from the Department for International Development

European Union

Consent

Hello, my name is ………………………………….. I am working in Health department/ family planning department as …………………………….. You may be happy to know that Directorate General of Health Services (DGHS) and Directorate General of Family Planning (DGFP) are working together in Thakurgaon district to identify and review all maternal and perinatal death which helps to improve health status in Bangladesh. As a part of this I would like to ask some questions and discuss with you about still birth. This interview will take 45 minutes approximately. This interview depends upon your wish, you may not answer all questions. Before you give your consent, you may ask any questions and know about the subject of interview. You don't have any risk to participate in the interview. All information related to this will keep confidential. Your name will not be mentioned in the report. The Ministry of Health and Family Planning will learn from your information and that will help to improve maternal and neonatal health status.
Thanks to you.

From above consideration, if you want to participate in interview, then I will start the session.

If respondent has given consent to participate in the interview:

Name of the respondent: ....

Signature of the respondent:..

Date of interview: | | | | | | |

Day / Month / Year

Interview starting time [In 24 hours] | | | | |

Interview end time [In 24 hours] | | | | |

Name of the interviewer:...................................... Designation.................Code no: | | |

Respondent's Infromation

Name	Relation with deseased	Sex	Religion	Race	Present during death of mother	When you last time saw (In days)
Principal respondent :						
Assocaite respodnet -1:						
Assocaite respodnet -2:						
Assocaite respodnet -3:						

<u>Relation:</u> 01. Father in law 02.Mother in law 03.Husband 04. Mother 05. Father 06. Grand father/ Grand mother 07. Grand father/ Grand mother (M) 08. dzdz/dzdv 09. Lvjv/Lvjy 10. PvPv/PvPx 11. gvgv/gvgx 12. Elder brother/sister 13. bb`/ fvex/ Rv 14. ‡`ei / fvmyi 15. Neigbourer 16. others (specify)..................................

<u>Sex :</u> 1. Male 2. Female **<u>Religion:</u>**1. Islam 2. Hindu 3. Buddist 4. christian **<u>Race</u>:** 1. Non-tribal 2. Tribal

<u>Presence :</u> 1. Yes 2. No

General Information

District..................☐Upazila.................. ☐ Union/Ward................................☐☐

Village: ....CC........................☐☐ Code No: ☐ ☐ ☐☐ ☐☐

District / Upazila/ Interviewer's code/ Still birth code

Mother's name: ....

Age of mother (In Years): ☐☐

Mother's Education: ☐☐

Husband's name:..

Age of husband (In years) : ☐☐

Husband's education : ☐☐

Education duration- (01. 02. 03........... 10........... 12 etc. and if no education then write code 99)

1. Date of death : ☐☐ ☐☐ ☐☐ Time (In 24 hours): ☐☐ ☐☐

   Day / Month / Year Hour / Minute

2. Did your fetus had movement during initiate of laobur pain? ☐☐

   01. yes 02. no 99. Not know

3. When did you last identified fetal movement ? (In hour/ minute) ☐☐ ☐☐

   Hour / minute

4. What was the colour of skin during still born ? ☐☐

   01. Normal (pink) 02. Blackish 03. Masserated 99. Not known

5. What was the size of still born? ☐☐

   01. Less than the normal size 02. Normal size 03

6. Sex of stillborn ☐☐

   01. Boy 02. Girl 03. Not understood 99. Not known

7. Accodring to statement, is there any complication occured during pregnancy? ☐☐

   (Multiple response)

   1. yes 2. no (Mention the underline complications)

(if no complications then go to ques. 12 and onwards)

a. High blood pressure

b. Diabetes

c. Antepartum haemorrhage

d. High fever

e. Swelling of hand, face and leg

f. Convulsion (Eclampsia)

g. Anaemia

h. Jaundice

i. Blurring of vision

j. Sudden unconciousness

k. No movement of fetus for logn duration

l. Other, specify....................................

8. Any treatment recevied during complication?

1. Yes 2. No

(if "no" then go to ques. 11 and onwards)

9. If answer is "yes" then where mother recevied treatment ? (Multiple response)

01. At home 02. Community clinic 03. Union health and family welfate centre

04. Upazila Health complex 05. Maternal and child welfare centere

06. Distrcit or sadar hosapital 07. Medical college hospital 08. Privat clinic

09. Privat hospital 10. NGO clinic 11. Chamber/ health provider's home

12. others (specify)...................................

10. Who treated the patinet ? (Multiple response)

01.Doctor (MBBS) 02. Nurse 03. Family welfare visitor (FWV)

04. Community skill birth attenddent (CSBA) 05. MA/ SACMO

06. Health assistant(HA) 07. Family welfare assistant (FWA)

08. Village doctor 09. NGO worker 10.Others (specify)...................................

11. If answer is no then why any treatment was not taken ? (Multiple response)

1. Mentioned 2. Not mentioned

a. Didnt think to treat

b. Didn’t know where had to go

c. Didn’t go beaucse it was so far away

d. Vehicle was not available

e. No one available to go was at that time

f. Time was not sufficient

g. Lack of available money

h. Barrier from the family

i. No one at home to take care of another child

j. It was not possible to go when the health care centre open

k. Was not possible to go due to Dark night/ natural disaster

l. Quality of care is not good

m. Behaviour of health care provider is not good

n. No one available in health care centre

o. others (specify)..

12. According to your statement why the still birth was occured?

1. Pregancny / Delivery related 2. trauma/ Injury ?

(If Pregancny / Delivery related then go to question 13)

12 (a). If death due to injury, specify?

………

13. Did the mother recieved antenatal care?

01. yes 02. no 99. Don’t know

(If answer is“no”or don’t know then go to ques. 17)

14. How many times received antenatal care? (Mention number)

15. From where antenatal care was received? (Multiple response)

01. At home 02. Community clinic 03. Union health and family welfate centre

04. Upazila Health complex 05. Maternal and child welfare centere

06. Distrcit or sadar hosapital 07. Medical college hospital 08. Privat clinic

09. Privat hospital 10. NGO clinic 11. Chamber/ health provider’s home

12. others (specify)...................................

16. Who provide antenatal care? (Multiple response)

01.Doctor (MBBS) 02. Nurse 03. Family welfare visitor (FWV)

04. Community skill birth attenddent (CSBA) 05. MA/ SACMO

06. Health assistant(HA) 07. Family welfare assistant (FWA)

08. Village doctor 09. NGO worker 10.Others (specify)...................................

17. Total number of delivered baby (para):

18. Total number of abortion (if not know/ applicable, write code 99)

19. How many months of preganncy at the time of still birth ? (In month)

(If don't know write code 99)

20. Place of death?

01. At home 02. Community clinic 03. Union health and family welfate centre

04. Upazila Health complex 05. Maternal and child welfare centere

06. Distrcit or sadar hosapital 07. Medical college hospital 08. Privat clinic

09. Privat hospital 10. NGO clinic 11. Chamber/ health provider's home

12. others (specify)...................................

21. Who delivered the fetus?

01.Doctor (MBBS) 02. Nurse 03. Family welfare visitor (FWV)

04. Community skill birth attenddent (CSBA) 05. MA/ SACMO

06. Health assistant(HA) 07. Family welfare assistant (FWA)

08. Village doctor 09. NGO worker 10.Others (specify)...................................

22. Mode of delivery?

1. Normal 2. Vacume 3. Foceps 4. Caesarean Section 5. others (specify)...............

23. Duration of labour ? (In hour / minute)

(From starting of labour pain upto deliver fetus) Hour / Minute

24. Any complications occured during delivery ?

01. yes 02. no 99. Don't know

(If answer is "no" or "not know" then go to question no. 25)

24 (a). What were those complications ? (Multiple response)

1. yes 2. no (Mention the underline complications)

a. Convulsions

b. Severe bleeding

c. Prolonged labour

d. Obstracted labour

e. Premature rupture of membrane

f. Abnormal presentation (hand, leg prolapse)

g. Retain Placenta

h. others (specify)...................................

25. Any complication occured after delivery ? (Multiple response)

1. yes 2. no (Mention the underline complications)

a. Haemorrhage

b. High fever

c. Jaundice

d. Convulsion (Eclampsia)

e. Sepsis

f. Rupture uterus

g. Retain placenta

h. Sudden unconciousness

l. Other, specify.....................................

26. Did mother recived any postnatal care?
01. yes 02. No 99. Not known/ not applicalbe (if delviery at hospital)

27. If yes, then how long after delviery (days/ hours)

28. From where mother recived post natal care ? (Multiple response)

01. At home 02. Community clinic 03. Union health and family welfate centre

04. Upazila Health complex 05. Maternal and child welfare centere

06. Distrcit or sadar hosapital 07. Medical college hospital 08. Privat clinic

09. Privat hospital 10. NGO clinic 11. Chamber/ health provider’s home

12. others (specify)....................................

29. Who provided postnatal care?

01.Doctor (MBBS) 02. Nurse 03. Family welfare visitor (FWV)

04. Community skill birth attenddent (CSBA) 05. MA/ SACMO

06. Health assistant(HA) 07. Family welfare assistant (FWA)

08. Village doctor 09. NGO worker 10.Others (specify)....................................
