# Supplementary figures and images for: Stillbirth surveillance and review in rural districts in Bangladesh
Source: BMC Pregnancy Childbirth. 2018 Jun 13;18:224. doi: 10.1186/s12884-018-1866-2 (PMC6004696; doi:10.1186/s12884-018-1866-2)

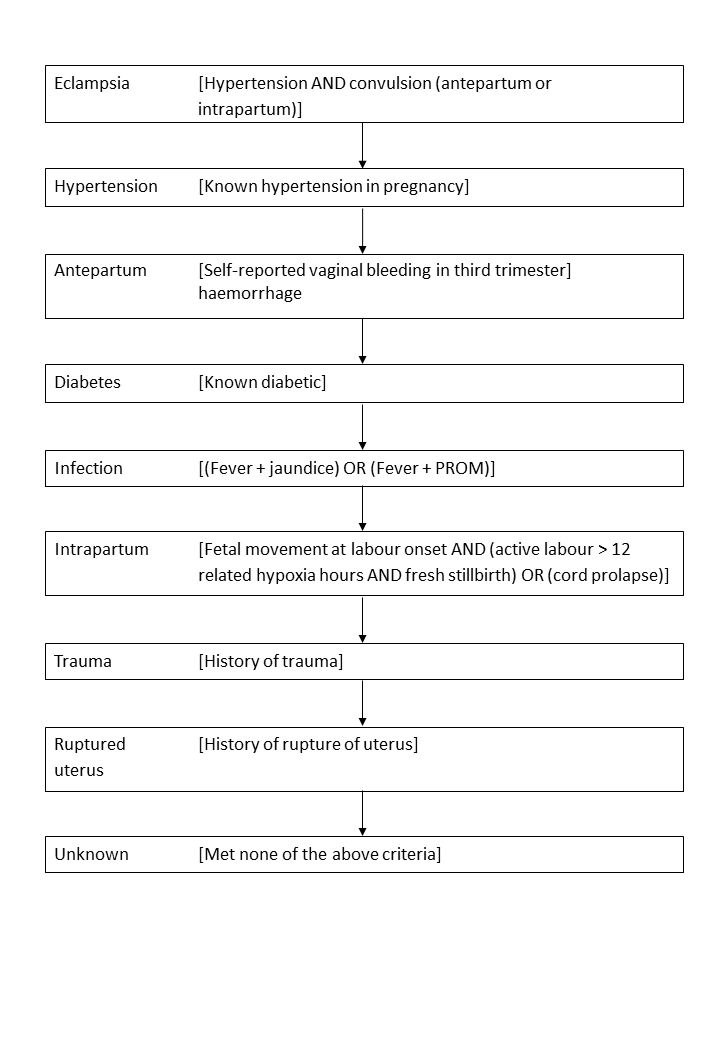

Supplement: Supplementary file 2 — Hierarchical model and criteria used to assign likely cause of stillbirth using information obtained via verbal autopsy. PROM = Premature rupture of membranes. (TIF 76 kb) [file 12884_2018_1866_MOESM2_ESM.tif]

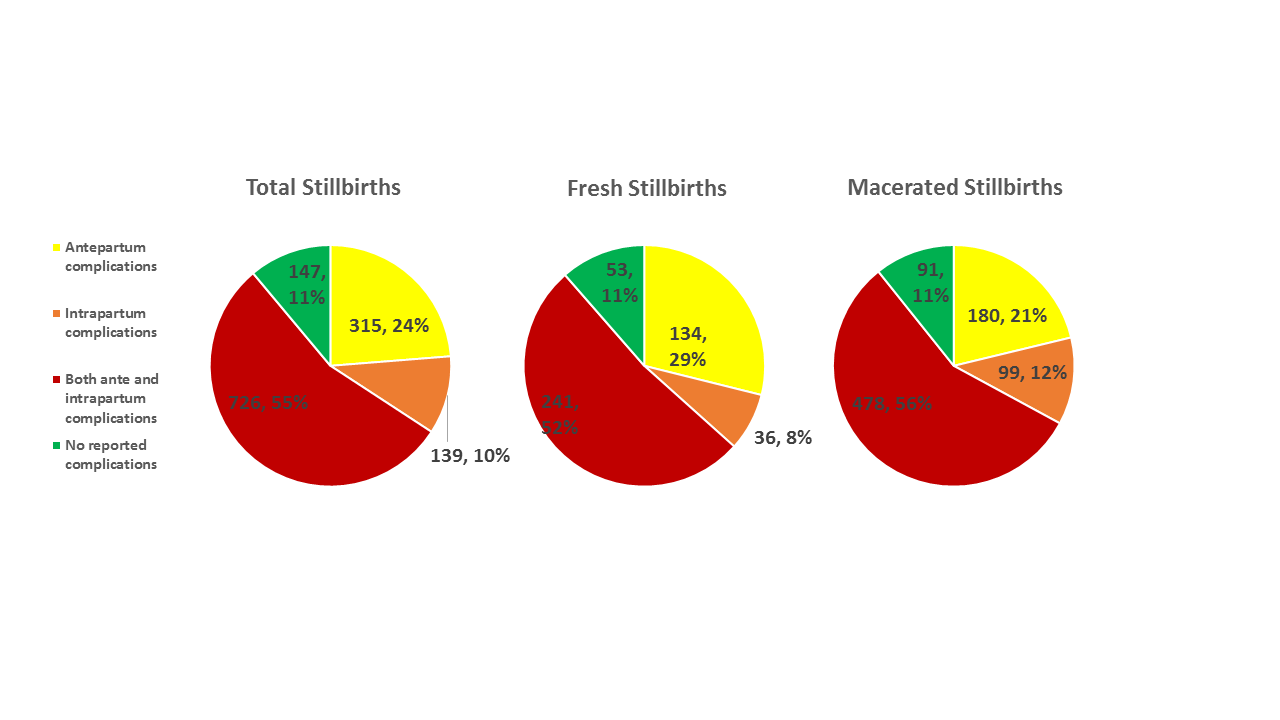

Supplement: Supplementary file 3 — Frequency of ante and intrapartum complications among mothers who experienced a stillbirth for all stillbirths combined, fresh stillbirths and macerated stillbirths (n = 1041). (TIF 95 kb) [file 12884_2018_1866_MOESM3_ESM.tif]
